# 2761. Activity of Cefiderocol and Comparator Agents Against *Achromobacter* and *Burkholderia* Isolates, Collected During 2020-2022 as Part of SENTRY Antimicrobial Surveillance Program

**DOI:** 10.1093/ofid/ofad500.2372

**Published:** 2023-11-27

**Authors:** Boudewijn L DeJonge, Sean T Nguyen, Jason J Bryowsky, Christopher M Longshaw, Joshua Maher, Rodrigo E Mendes, Miki Takemura, Yoshinori Yamano

**Affiliations:** Shionogi Inc., Florham Park, New Jersey; Shionogi Inc., Florham Park, New Jersey; Shionogi Inc., Florham Park, New Jersey; Shionogi B.V., London, England, United Kingdom; JMI Laboratories, North Liberty, Iowa; JMI Laboratories, North Liberty, Iowa; Shionogi & Co., Ltd, Toyonaka, Osaka, Japan; Shionogi & Co., Ltd., Toyonaka, Osaka, Japan

## Abstract

**Background:**

*Achromobacter* and *Burkholderia* species are opportunistic pathogens in individuals with an immunodeficiency, most notably cystic fibrosis. Cefiderocol (CFDC) is a siderophore-conjugated cephalosporin with broad activity against Gram-negative bacteria. In this study, the activity of CFDC and comparator agents was determined against isolates of *Achromobacter* and *Burkholderia cepacia* species complex, collected in 2020–2022 in Europe and the USA as part of the SENTRY antimicrobial surveillance program.

Activity of cefiderocol and comparator agents against Achromobacter and Burkholderia Isolates

**Figure:**
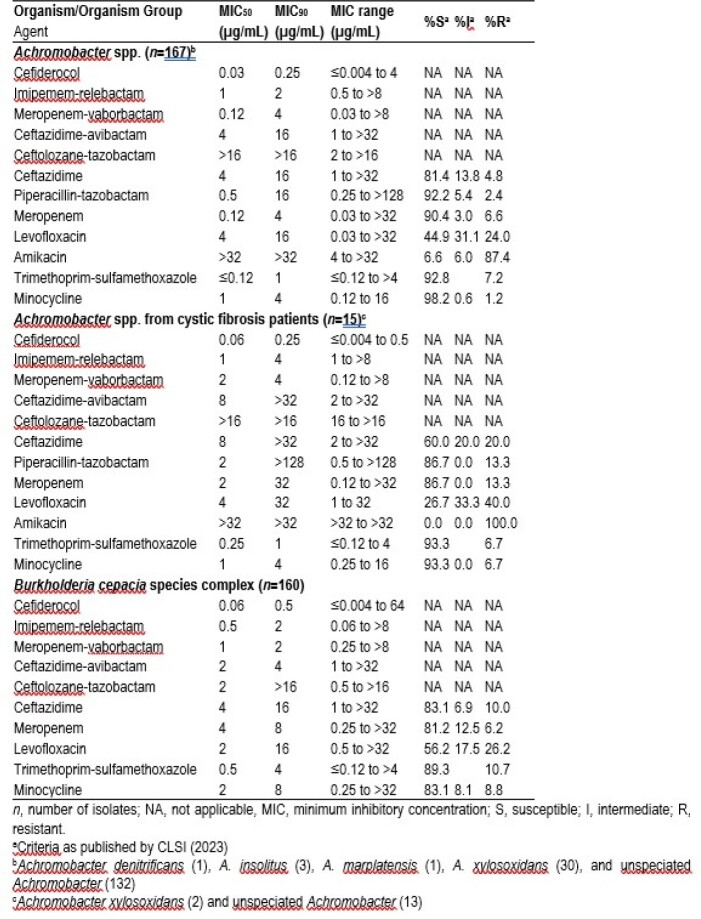

**Methods:**

Minimum inhibitory concentrations (MICs) were determined according to CLSI guidelines against 167 *Achromobacter* and 160 *Burkholderia cepacia* species complex isolates, using broth microdilution with cation-adjusted Mueller-Hinton broth (CAMHB) for comparator agents and iron-depleted CAMHB for CFDC. Comparator agents included β-lactam/β-lactamase inhibitor combinations ceftazidime-avibactam, ceftolozane-tazobactam, imipenem-relebactam, meropenem-vaborbactam, piperacillin-tazobactam, as well as meropenem, ceftazidime, levofloxacin, amikacin, trimethoprim-sulfamethoxazole, and minocycline. Susceptibility was assessed for agents with CLSI breakpoints. For agents without established CLSI breakpoints, only MIC_50_, MIC_90_ and MIC ranges were reported.

**Results:**

CFDC was the most potent agent tested against *Achromobacter*, showing MIC_50_ and MIC_90_ values of 0.03 and 0.25 µg/mL and all isolates were inhibited at ≤4 µg/mL (Table). Isolates from cystic fibrosis patients (n=15) showed higher MIC_50_ and MIC_90_ values for most agents, but CFDC MIC_50_ and MIC_90_ values remained low (0.06 and 0.25 µg/mL, respectively). Against *Burkholderia cepacia* species complex, CFDC was the most potent agent tested, with MIC_50_ and MIC_90_ values of 0.06 and 0.5 µg/mL, and 156 out of 160 isolates inhibited at ≤4 µg/mL. None of the comparator agents showed >90% susceptibility against these isolates (Table).

**Conclusion:**

CFDC showed potent activity against a set of contemporary clinical *Achromobacter* and *Burkholderia cepacia* species complex isolates. These *in vitro* data suggest that CFDC could be an important treatment option for infections caused by these opportunistic pathogens.

**Disclosures:**

**Boudewijn L. DeJonge, PhD**, Shionogi Inc.: Employee **Sean T. Nguyen, PharmD**, Shionogi: Employee|Shionogi, Inc: Employee **Jason J. Bryowsky, PharmD, MS**, Shionogi Inc.: Employee **Christopher M. Longshaw, PhD**, Shionogi BV: Employee **Joshua Maher, PhD**, AbbVie: Grant/Research Support|Affinity Biosensors: Grant/Research Support|AimMax Therapeutics, Inc: Grant/Research Support|Alterity Therapeutics: Grant/Research Support|Amicrobe, Inc: Grant/Research Support|Arietis Pharma: Grant/Research Support|Armata Pharmaceuticals, Inc: Grant/Research Support|Astrellas Pharma, Inc.: Grant/Research Support|Basilea Pharmaceutica AG: Grant/Research Support|Becton Dickinson And Company: Grant/Research Support|bioMerieux, Inc: Grant/Research Support|Boost Biomes: Grant/Research Support|Diamond V: Grant/Research Support|Fedora Pharmaceuticals, Inc: Grant/Research Support|Iterum Therapeutics plc: Grant/Research Support|Johnson & Johnson: Grant/Research Support|Kaleido Biosciences, Inc.: Grant/Research Support|Meiji Seika Pharma Co. Ltd.: Grant/Research Support|National Institutes of Health: Grant/Research Support|Pfizer Inc.: Grant/Research Support|Roche Holding AG: Grant/Research Support|Shionogi Inc.: Grant/Research Support|Summmit Therapeutics, Inc.: Grant/Research Support|Zoetis Inc: Grant/Research Support **Rodrigo E. Mendes, PhD**, AbbVie: Grant/Research Support|Basilea: Grant/Research Support|Cipla: Grant/Research Support|Entasis: Grant/Research Support|GSK: Grant/Research Support|Paratek: Grant/Research Support|Pfizer: Grant/Research Support|Shionogi: Grant/Research Support **Miki Takemura, n/a**, Shionogi & Co., Ltd.: Stocks/Bonds **Yoshinori Yamano, PhD**, Shionogi HQ: Employee

